# Water Polishing improved controlled-release characteristics and fertilizer efficiency of castor oil-based polyurethane coated diammonium phosphate

**DOI:** 10.1038/s41598-020-62611-w

**Published:** 2020-04-01

**Authors:** Hao Lu, Hongyu Tian, Min Zhang, Zhiguang Liu, Qi Chen, Rui Guan, Huaili Wang

**Affiliations:** 1 National Engineering Laboratory for Efficient Utilization of Soil and Fertilizer Resources, National Engineering and Technology Research Center for Slow and Controlled Release Fertilizers, College of Resources and Environment, Shandong Agricultural University, Taian, Shandong 271018 China; 2 State Key Laboratory of Nutrition Resources Integrated Utilization, Kingenta Ecological Engineering Group Co., Ltd., Linshu, 276700 China

**Keywords:** Chemical physics, Chemical engineering

## Abstract

The production cost of controlled-release fertilizers is an important factoring limiting their applications. To reduce the coating cost of diammonium phosphate (DAP) and improve its nutrition release characteristics, the fertilizer cores were modified by water polishing with three dosages at 1, 2, and 3%. The effects of modification were evaluated in terms of particle hardness, size distribution, angle of repose and specific surface area. Castor oil-based polyurethane was used as coating material for fertilizer performance evaluation. A pot experiment was conducted to verify the fertilizer efficiency of coated diammonium phosphate (CDAP) with maize. The results showed that polishing with 2% water reduced the angle of repose by 2.48–10.57% and specific surface area by 5.70–48.76%, making it more suitable for coating. The nutrient release period of CDAP was significantly prolonged by 5.36 times. Soil available phosphorous, enzyme activities, maize grain yield, and phosphorous use efficiency were all improved through the blending application of coated and normal phosphate fertilizer. This study demonstrated that water-based surface modification is a low-cost and effective method for improvement and promotion of controlled release P fertilizers.

## Introduction

Phosphorus (P) is an essential nutrient for maintaining healthy crop growth, reproduction and yield^1–3^. However, farmers tend to apply excessive amount of P fertilizers in order to obtain high yields^4^, resulting in degradation of microbial community activity and soil tilth^5^, decrease in crop quality^6^, and varying forms of water pollution^7^. Meanwhile, P fertilizers are mainly derived from P rock, a non-renewable resource. With the increasing use of P fertilizer, the global P reserve is facing serious concerns^8^. Innovative technologies for improving the utilization efficiency and reducing the production cost of P fertilizers are urgently needed.

Controlled-release fertilizers have been proved to improve the utilization efficiency of both nitrogen (N) and P fertilizer^9,10^. While much research has been conducted on controlled-release N fertilizers^11,12^, relatively much less work has focused on controlled-release P fertilizers. Chemically bound P in soil could become available to plant under certain biogeochemical conditions, serving as a natural reservoir of “slow-release” P^13^. However, the slow release characteristics of soil bound P are affected by many uncontrollable factors, such as temperature, humidity, organic acid secreted by crop roots, soil pH, and soil mineral composition^14–17^. Thus, P released under these conditions cannot fulfill the crop P demand, especially during the critical growth period. Recent research indicated that the fixed rate of soil P was closely related to the concentration of P in soil solution^18^. Coated P fertilizers have advantages over conventional P fertilizers in that the polymer coating prevents direct contact between soil and fertilizer, thus the controlled release of P promotes uptake by plants and reduces P fixation by soil^19^.

While the controlled-release P fertilizer has been noted to improve crop yield and P use efficiency (PUE)^20,21^, its wide application was limited partly because of the difficulty in controlling release characteristics with crop P requirement^22,23^. The nutrient release rate can be controlled by adjusting the amount of coating materials. da Cruz^24^ improved the release characteristics of coated diammonium phosphate (CDAP) through the use of bio-based polyurethane as coating material. However, the amount of membrane material he used in the experiment was as high as 9%, leading to high cost, which limited the promotion of CDAP. Several attempts also started with the modification of membrane materials to improve the controlled-release characteristics^25–27^. Zhang and Xie prepared superhydrophobic surface with organosilicon modified bio-based polyurethane to prolong the release period of coated fertilizer^25,26^. Xie improved the controlled-release characteristics with magnetic nanomaterials^27^. It is undeniable that their work achieved desired results, yet the synthesis of coating materials were complex and costly. There were no scientific guidelines compatible with the application of controlled-release P fertilizers.

In the coating process of fertilizer, particles with regular shape and smoother surface usually have high film forming rate, resulting in good controlled-release performance^28^. Therefore, the quality of fertilizer core also plays a very important role in the preparation and production of coated fertilizer. However, little research has been conducted to examine how the fertilizer core can be optimized to enhance the controlled release characteristics. This study attempts to fill in this information gap by improving the shape and surface properties of fertilizer core with a novel water polishing technique.

Polishing process is an effective method in industrial production for improving the surface properties and shape of materials^29,30^. During the polishing process, medium materials such as inert gas and surfactant are usually added to achieve functionalities like lubrication and protection^31,32^. Compared with the mediums mentioned above, water is a simpler and more accessible material, which may serve as a moisturizing and buffering agent during the polishing process.

We hypothesize that water-based surface modification is effective in improving the performance of controlled release P fertilizer while reducing its production cost. The objectives of this study are to 1) estimate the effect of water polishing on DAP particle surface and fluidization; 2) explore the fertilizer effect of CDAP on maize yield with a pot experiment.

## Materials and Methods

### Materials

The diammonium phosphate (DAP) was purchased from Yunnan Yuntianhua Co., Ltd. (Kunming, China), and the particles with diameters of 3–5 mm were used for water polishing and coating. Polyaryl polymethylene isocyanate (PAPI) with 30.03 wt% NCO groups was provided by Yantai Wanhua Polyurethane Co., Ltd. (Yantai, China). Castor oil (Hydroxyl value = 167.3 mg KOH g^−1^) was purchased from Yi Hai Oil Industry Co., Ltd. (Yantai, China). Maize (*Zea mays L*. ‘Zhengdan 958’) was used for the fertilizer efficiency test, purchased from Shandong Denghai Seeds Co., Ltd. (Taian, China). Urea (46% N), potassium chloride (60% K_2_O) and diammonium phosphate (DAP, 18%N, 46% P_2_O_5_) were obtained from local fertilizer distributors. The soil for pot experiment was taken from the research farm of Shandong Agricultural University where no P fertilizer was applied for four consecutive years. The physical and chemical properties of the soil were: pH (7.51 with soil to water ratio 1:2.5), available phosphorus (14.83 mg kg^−1^), organic matter (11.81 g kg^−1^), total N (0.70 g kg^−1^), NO^3−^-N (19.39 mg kg^−1^), NH^4+^-N (11.24 mg kg^−1^), and available potassium (88.36 mg kg^−1^).

### Preparation of modified DAP and CDAP

**Figure 1:**
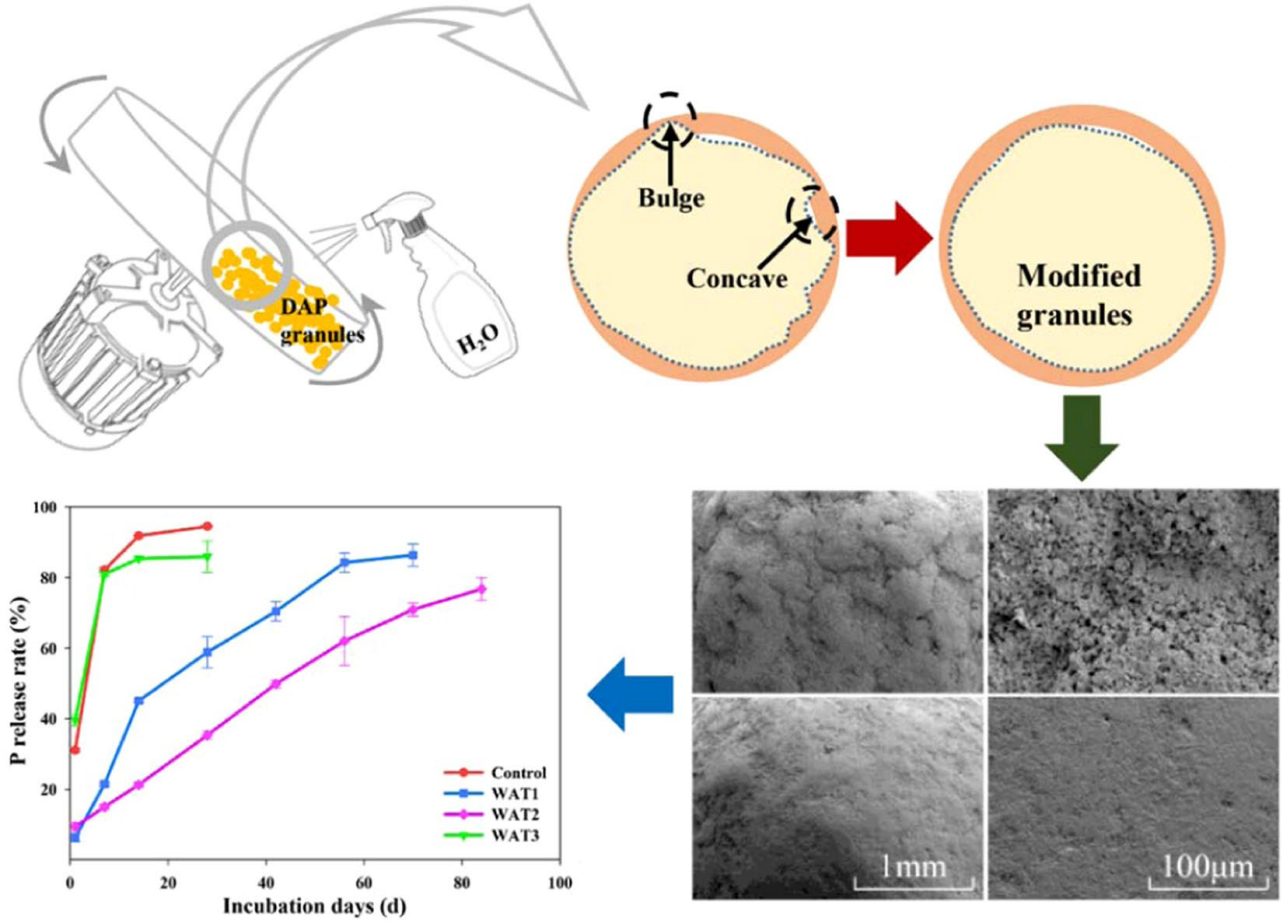
Preparation and mechanism of modified DAP and CDAP.

Modification of DAP and preparation of CDAP were carried out in a rotating drum designed by our laboratory and produced by Shandong Hongtai Instrument Co., LTD (Tai-an, China). The experiment was designed in Fig. 1. Water was chosen as modified materials, which was aimed to polish the surface bulges of the particles. One kilogram of DAP was weighed each time, when the fertilizer was heated to 50 °C, sprayed the water to the surface of fertilizer particles through a sprayer. The depth and diameter of the drum were 45 cm and 50 cm, respectively. During the whole process, the inclination angle and the rotation speed of the drum was 30°and 40 RPM, respectively. The treatment code and details were given as follows (Table 1).

**Table 1:** Treatment code and details.

Code	Treatment	Initial temperature (°C)	Final temperature (°C)	Loss (%)
Control	untreated	—	—	—
WAT1	DAP + 1% water	50	65	0.69
WAT2	DAP + 2% water	50	65	0.81
WAT3	DAP + 3% water	50	65	1.07

Castor oil and PAPI were used as membrane material with a ratio of 6:4. First, 1-kilogram fertilizer was heated to 65 °C. A mixture of 6.0 g castor oil and 4.0 g PAPI were then poured on the fertilizer particle surface. The amount of coating materials added each time was 1% of the weight of fertilizer. The coating procedure was repeated 2, 3 and 4 times to obtain three more coating contents (2, 3, 4%)^20–24^.

### Characterization

Field emission scanning electron microscopy (SEM, Model JSM-7500F, Japanese Electronics Corp., Japan) was used to observe surface morphology and smoothness. Angle of repose (AOR) was determined using an AOR tester (FBS-104, FURBS, China) with 100 g DAP particles of different treatments poured into the tester. The height of the particle pile (h, cm) and the radius of bottom tray (5 cm) were recorded for calculation of the angle of repose (α) as below: α = Arctan (h/5). Particle roundness was tested through a roundness tester (Winner 300D, Winner particle instrument Co. Ltd, Jinan, China). A particle hardness tester (Fangyuan test instrument Co. LTD, Jinan, China) was used to test particle crushing strength by applying an increasing pressure on a single particle. The tester would record the pressure when the particle was crushed. For each treatment, 20 particles were randomly sampled from the final product. In this experiment, Newton (N) was used as the unit of particle hardness. Kr adsorption was carried out to measure the specific surface area (SSA) with an SSA and pore size analyzer (JW-BK300C, Beijing, China) at −196 °C^33^.

The CDAP release characteristics was determined by static water extraction: 10.00 g of CDAP was weighed each time, placed in a gauze bag and soaked in a glass bottle with 200 mL water. The bottles were placed in a 25 °C incubator. 10 mL water of each bottle was sampled at 1, 7, 14, 28, 42, 56, 70, 84 and 98 days after incubation for measurement of P concentration until the accumulative release rate reached 80%^34^.

### Pot experiment

Four treatments were carried out to verify the fertilizer efficiency of CDAP: (1) without phosphate fertilizer (P0); (2) DAP (P1); (3) 40% CDAP with 60% DAP (C40P1); and (4) 60% CDAP with 40% DAP (C60P1). The amount of phosphate fertilizer applied to all treatments with phosphate fertilizer was 3.2 g P_2_O_5_ pot^−1^. The CDAP used in the pot experiment was WAT2 with 3% coating materials. Except for DAP and CDAP, the rest of nitrogen and phosphate fertilizer was supplied by common urea and calcium superphosphate, respectively. The nitrogen application amount of all treatments was 3.2 g N pot^−1^. The application rate of potassium is 1.6 g K_2_O pot^−1^ as potassium chloride. All fertilizers were mixed evenly in 15 kg soil and put into a pot with 10 kg sand at the bottom.

Auxin (IAA), gibberellin (GA), phosphoenolpyruvate carboxylase (PEPC), ADP-glucosepyrophosphorylase (AGPase), phosphoribosyl pyrophosphate (PRPP), adenosine monophosphate synthetase (AMPSS) and phosphoribosyl pyrophosphate aminotransferase (PRPPAT) were extracted and assayed with a ELISA kit from Shanghai HengYuan Biological Technology Co. Ltd. (Shanghai, China). Soil available P content was extracted with 0.5 mol L^−1^ sodium bicarbonate solution, and the absorbance was measured by spectrophotometer after molybdenum and antimony^35^. Phosphorus use efficiency (PUE) = (P accumulation in P area - accumulation of P in blank area)/P application rate × 100%^36^.

### Statistical analysis

Data analysis was carried out with SAS (version 9.2, SAS Institute, Cary, NC). The differences among means and correlation coefficients were considered significant when *P* < 0.05. Sigmaplot (version 14.0, Systat Software Inc.) and Photoshop CS6 (Adobe Systems Inc.) were used for preparation of figures.

## Results and Discussion

### Surface morphologies of modified DAP

**Figure 2:**
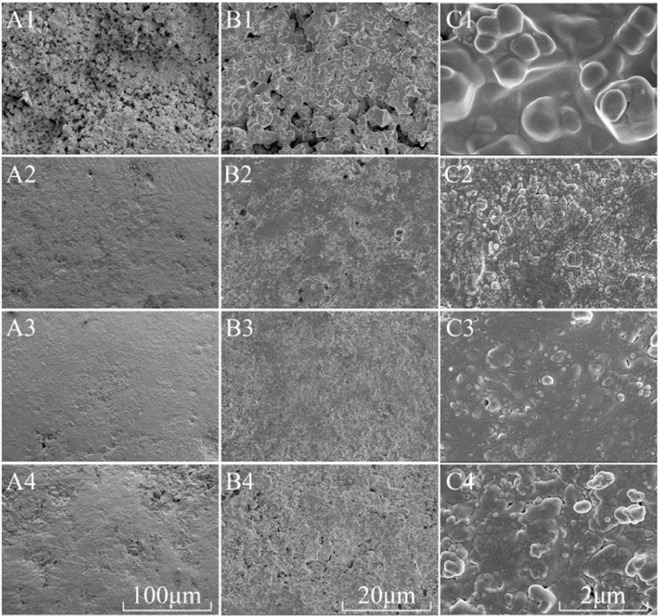
Particle surface of different treatments, panels A1, A2, A3, and A4 show the surface of Control, WAT1, WAT2, and WAT3 treated particles, respectively. The image magnification is 500×. Panels B1, B2, B3, and B4 show the surface of Control, WAT1, WAT2, and WAT3 treated particles, respectively. The image magnification is 2000×; panels C1, C2, C3, and C4 show the surface of Control, WAT1, WAT2, and WAT3 treated particles, respectively. The image magnification is 20000×.

In this study, DAP particles were modified with water. Physically, it was a dissolve-recrystallization process, which was generally difficult to change the crystal structure of DAP, and this could be seen dimly from high-power electron microscopy (Fig. 2). Therefore, the effect of water polishing on the surface of fertilizer particles was studied in this paper, but did not involve the crystal growth. The surface morphology of DAP particles was observed by scanning electron microscopy (SEM). The surface of unmodified DAP particles exhibited raised and sunken micro-features (Fig. 2A1, A2), which were smoothed after water polishing (Fig. 2B1, B2, C1, C2). In this process, the bulges on DAP particle surfaces were softened, dissolved, and then re-granulated, similar to the third stage of wet granulation^37^. Of all the surface modification treatments, WAT2 had the best effect, suggesting that water added in WAT1 was so little that it would evaporate soon after spraying onto the fertilizer while the amount of water added in WAT3 was excessive and caused particle aggregation. The regular and smooth surface of modified DAP can decrease the contact area between the membrane material and the surface of fertilizer particles, thereby improving the membrane structure.

### The fluidized characteristics of the modified particles

Roundness was measured to characterize the fluidization of DAP particles. Water polishing improved the roundness of DAP particles, although this effect was not significant in statistics (Table 2). Compared with the Control treatment, roundness of polished particles increased by 0.11–1.53%.

**Table 2:** Particle roundness and SSA of different treatment.

Treatment	Roundness	Change VS Control (%)	SSA (m^2^/g)	Change VS Control (%)
Control	0.918	—	0.02139	—
WAT1	0.931	1.42	0.01288	−39.78
WAT2	0.932	1.53	0.01096	−48.76
WAT3	0.909	0.11	0.02017	−5.70

Water-based polishing had a very significant effect on the SSA of DAP particles (Table 2). The SSA decreased by 39.78–48.76% when 1% to 2% water was added. However, compared with Control treatment, the SSA of WAT3 treatment decreased by only 5.70%. The reason may be that excessive water addition makes the surface of fertilizer particles stickier and increases the friction between particles. As a result, the particles rolled too slowly to be polished. The SSA of DAP particles was closely related to the amount of coating materials and the cost. In general, the quantity of raised and sunken structures on particle surface determines the SSA of DAP particles. Given the same particle mass, the smaller the SSA, the less the coating material would be used^38^.

**Figure 3:**
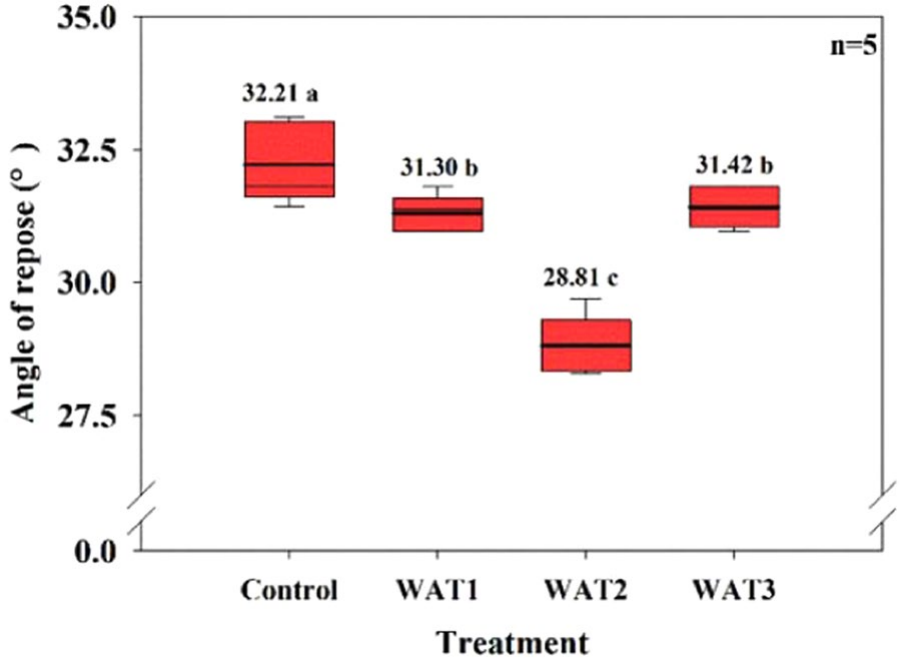
Angle of repose of different modified treatments.

NOTES: Each box includes data for the data set. The thicker solid lines within the box represent the means, and the thinner solid lines represent the medians; the lower and upper whiskers represent upper-lower limits of data, respectively; and the lower and upper boundaries of the boxes are 25th and 75th percentiles, respectively. For each factor, means followed by a same lowercase letter in the same column was not significantly different by Duncan’s test (*P* < 0.05).

The AOR of granular material is the inclination angle relative to the horizontal plane when the particle is at static condition. At this angle, the material on the slope is at the edge of sliding^39^. Generally, the decrease of AOR is indicative of improved fluidization characteristics of particles. The measured AOR of different treatments were shown in Fig. 3. Compared with Control treatment, the AOR of the particles after polishing was significantly reduced, with a decline of 2.48–10.57%, among which WAT2 treatment had the most significant effect. With the decrease of AOR, particles could roll more smoothly in the process of coating. This would increase the contact between particles and membrane materials, and eventually improve the membrane formation rate.

### Particle diameters

**Figure 4:**
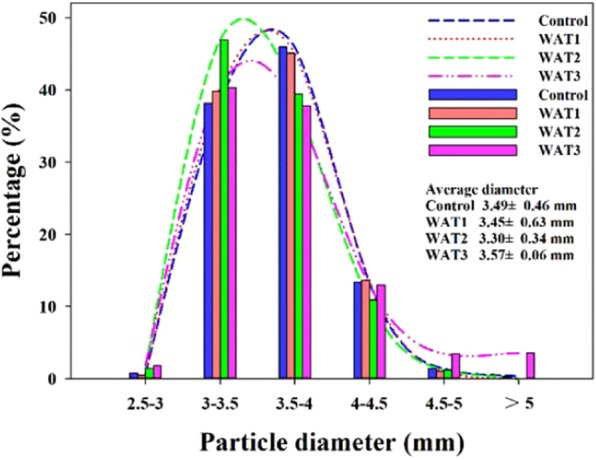
Particles size distribution of different treatment.

The fertilizer particle diameters of different treatments were shown in Fig. 4. In WAT2 treatment, the particle size of 3–3.5 mm increased significantly, while in WAT3 treatment, the particles larger than 4.5 mm in diameter increased significantly. Suitable amount of water (WAT2) softened the surface of fertilizer particles and played a key role in re-melting and re-engineering during the rotation process. However, too much water (3%) would cause excessive humidity of fertilizer particles, which led to adhesion and poor quality of fertilizer particles. This finding has a great implication in large-scale industrialized production.

### Membrane structures

**Figure 5:**
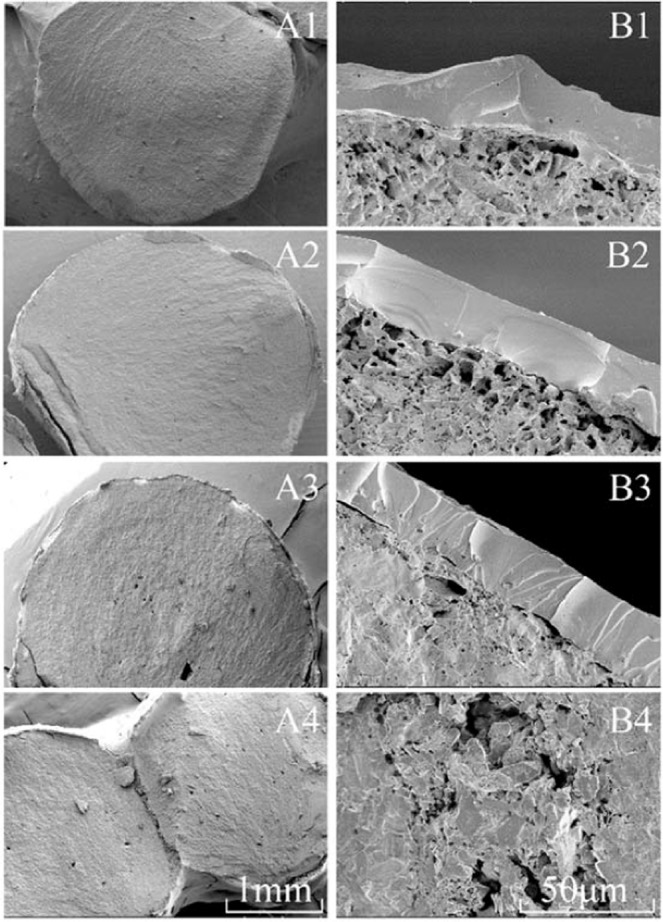
SEM images with 30× (A1, A2, A3, A4) and 1000× (B1, B2, B3, B4) magnification of Control, WAT1, WAT2, and WAT3 treated particles, respectively.

The film thickness of the Control treatment was not uniform (Fig. 5B1), while that of WAT1 and WAT2 treatments was much more regular (Fig. 5B2, B3). During the coating process, the raised and sunken structures on the particle surface tended to cause uneven film formation, such as exposing at the raised spot and accumulation of coating materials at the sunk spot. When particle surface became smoother with water polishing, the particles were in closer contact with the membrane material, and the unnecessary use of membrane material described above were reduced. Thus, water polishing allowed the coating material to be sprayed on the particle surface more evenly, thereby resulting in uniform film thickness^40^. Note that the asymmetrical membrane structure was formed at the uneven adhesion site of DAP particle (Fig. 5A4), likely because excessive water soaked the fertilizer surface and formed a “liquid bridge”^41^. When being steamed and dried, the “liquid bridge” cooled and solidified to a “solid-bridge”^42^. This led to the situation in Fig. 5B4, where DAP particles were tightly bonded together, affecting the formation of coating.

### Particle hardness

**Figure 6:**
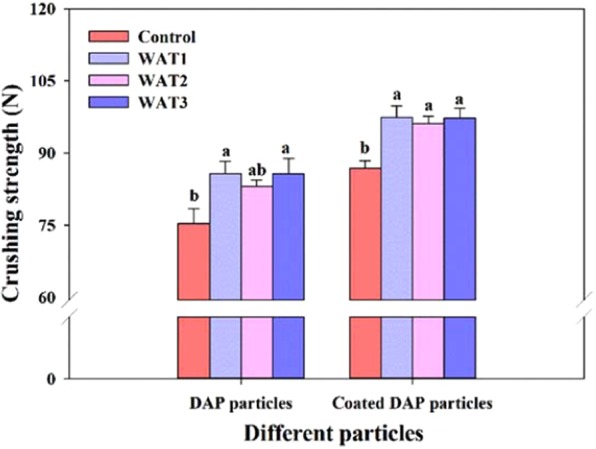
Hardness of DAP and CDAP particles under different treatments. Notes: Bar heights represent means and error bars represent ± SE. The same letters on the bars of each different particle (DAP particles and CDAP particles) were not significantly different based on one-way ANOVA followed by Duncan’s multiple-range tests (P < 0.05).

Particle hardness plays an important role in the coating process^28^. Polishing with water effectively improved DAP and coated DAP particle strength with an increase in particle hardness by about 12% (Fig. 6). The increase of particle hardness may be due to the decrease of porosity on the particle surface and the more regular shape of the particles^43^. When particles were extruded, the forces acting on the particles with more regular shapes were balanced and less likely to be broken^44^. Note that no significant difference was found between WAX treatments, suggesting at WAX1 was effective enough in improving particle strength.

### Nutrient release rate of CDAP with different coating content

**Figure 7:**
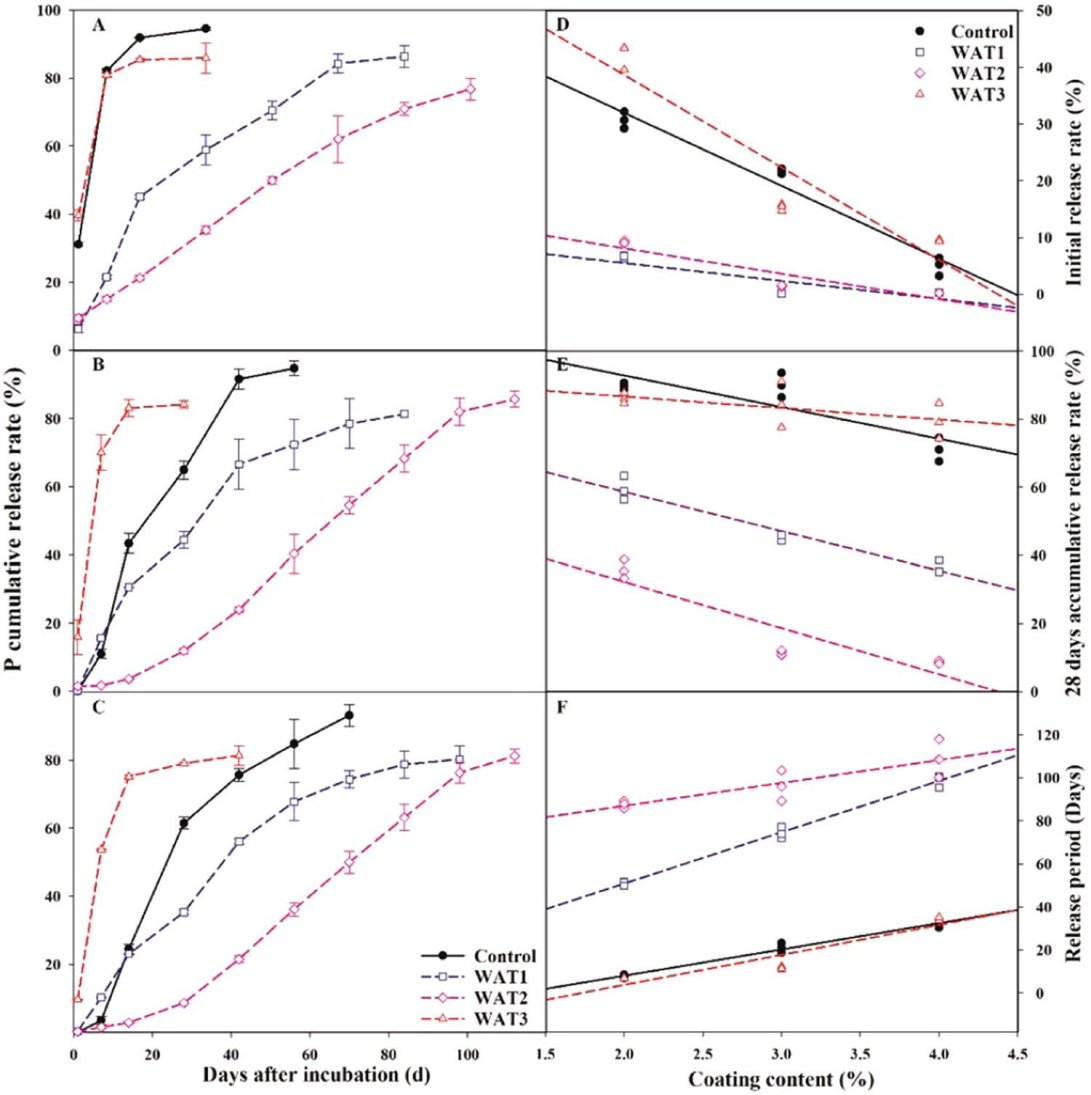
Phosphorus release characteristic of CDAP with different coating contents: 2% (**A**), 3% (**B**), and 4% (**C**), and the relationship between initial release rate (**D**), 28 days accumulative release rate (**E**), release period (**F**) and coating content, error bars represent ± SE.

The release period of fertilizer was significantly extended with the increase of the coating content (Fig. 7). For the Control, the initial release rate decreased from 31.07% to 5.84% and the P release period prolonged from 6.8 days to 29.7 days as the coating material increased from 2% to 4%. Water polishing made the effect of coating more effective. That is especially true for WAT2 whereby the initial release rate was reduced to 0.22%, and the release period was extended to 108.5 days with 4% coating content. The release curve of CDAP was close to the ideal ‘S’ shape, reflecting the effect of smaller AOR and SSA on the regularity of membrane.

Figure 7 D, E, F showed the fitting curve of coating content and initial release rate, 28 days accumulative release rate and release period. Compared with the conventional CDAP, the initial release rate of modified CDAP decreased by 4.61–24.04%. According to the fitting equation, when the initial release rate was less than 5%, the minimum coating content required by Control, WAT1, WAT2 and WAT3 treatment was 4.09%, 2.16%, 2.69% and 4.06%, respectively. The WAT1 and WAT2 treatments performed the best. Water polished CDAP could save coating quantity by more than 50%, in comparison to the common CDAP. The reduction of coating material consumption could accelerate the coating process, reducing labor costs and energy consumption.

### Grain yield and PUE of different fertilization treatments

Blended application of CDAP and normal DAP significantly increased the yield and PUE of potted maize (Table 3). Similar to coated urea and potassium chloride which significantly improve crop yield and nutrient use efficiency^45–47^, CDAP/DAP application increased grain yield and net profit by 8.14–23.69%, 24.40–85.58%, respectively. This is likely because the nutrient release characteristics of coated phosphate fertilizer matches nutrient uptake requirement of maize much better than conventional P fertilizer^48^. Also note that the yield of C40P1 was higher than that of C60P1, suggesting that the application ratio of coated phosphate fertilizer should not be too high.

**Table 3:** Grain yields of maize, PUEs and net profit of different fertilization treatments.

Treatment	Grain yield of maize (g/pot)	PUE (%)	Income ($/hm^2^)	Fertilizer costs ($/hm^2^)	Labor cost ($/hm^2^)	Net profit ($/hm^2^)	Change relative to P1 (%)		
							Grain yield	PUE	Net profit
P0	113.7 cd	—	2692.72	643.70	72.5	656.02	−8.38	—	−15.80
P1	124.1 c	19.6	2939.02	766.84	72.5	779.17	—	—	—
C40P1	153.5 a	38.0	3635.28	796.33	72.5	1445.95	23.69	93.88	85.58
C60P1	134.2 bc	31.7	3178.21	815.93	72.5	969.28	8.14	61.73	24.40

Note: The maize yield per hectare was calculated as 83325 maize plants. Materials costs were calculated according to the local price. The price of maize grain was 284.22 $/ton, CDAP was 540.28 $/ton, urea was 238.80 $/ton, labor cost for one fertilization was 72.5 $/hm^2^, other costs (1320.5 $/hm^2^) included irrigation, pesticides, seeds and other materials and expenses during the maize growth season.

### Effects of blending application of fertilization on soil available P content

**Figure 8:**
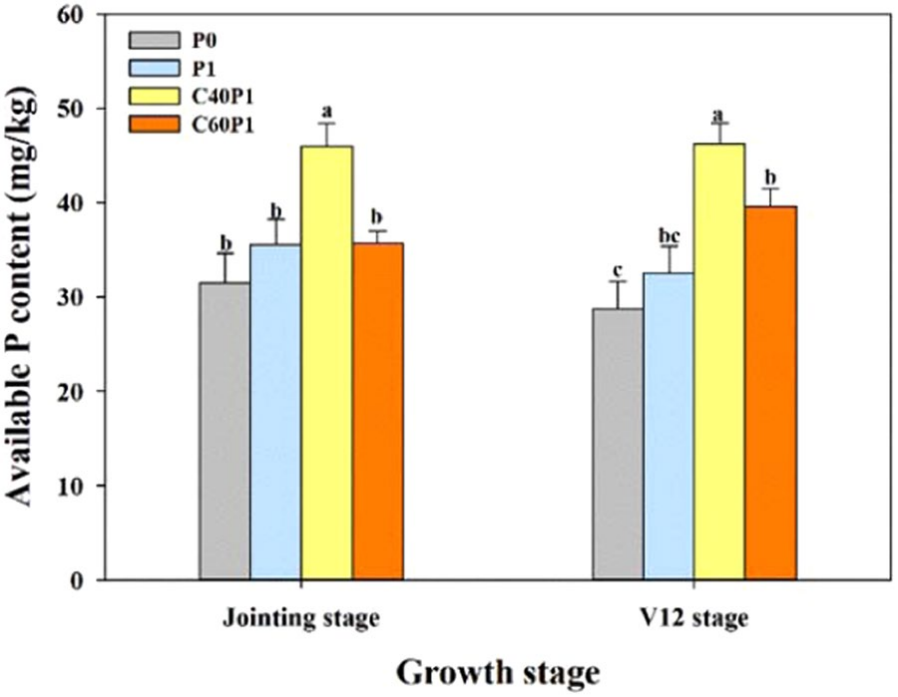
Soil available P content in two growth stages of maize.

Different fertilization treatments had significant effect on soil available P content in the two growth stages of maize (Fig. 8). At the jointing stage of maize, the available P content of the C40P1 treatment was 31.49% higher than that of P1 treatment. However, there was no significant difference among the other three treatments. At the V12 stage of maize, the soil available P content of P1 treatment was higher than that of P0 treatment, although there was no statistically significant difference. The soil available P content of the C40P1 and C60P1 treatment increased by 21.36% and 12.95%, respectively, over P1 treatment.

### Effects of blending application of DAP and CDAP on plant enzymes and endogenous hormones

Plant enzymes and endogenous hormones play a regulatory role in plant growth^49^. PEPC and AGPase were two important enzymes in the photosynthesis (Table 4). The order of activity of PEPC and AGPase in different treatments was C40P1 > C60P1 > P1, indicating that the application of blending phosphate fertilizer could significantly improve the activity of PEPC and AGPase and promote the photosynthesis of maize plants. PRPP, PRPPAT, AMPSS are important in nucleotide metabolism. Their activities were similar to that of PEPC and AMPSS, indicating that CDAP improved the metabolic rate of plants. IAA and GA both promote plant growth. The activities of IAA and GA followed the order of P0 treatment > ordinary phosphate fertilizer treatment > blending phosphate fertilizer treatment. This result was expected because maize produces more IAA and GA to promote its growth under P stress^50^.

**Table 4:** Several plant enzymes and endogenous hormones of leaves at V12 stage under different treatment.

Treatment	PEPC (U/g)	AGPase (U/g)	PRPP (U/L)	AMPSS (U/L)	PRPPAT (U/L)	IAA (U/L)	GA (ng/g)
P0	0.21 c	254.52 d	150.35 d	263.99 c	127.60 c	51.70 a	4.74 a
P1	0.17 d	384.35 b	200.81 c	333.85 b	131.85 b	46.21 b	4.05 b
C40P1	0.27 a	398.35 a	245.49 a	371.73 a	139.65 a	35.13 d	3.82 c
C60P1	0.25 b	344.18 c	216.10 b	269.88 c	128.30 bc	40.54 c	3.55 d

## Conclusions

We can now conclude that WAT2 performed the best among all the treatments. When 2% water was added for water polishing, the crushing strength of DAP particles increased by 10.19% while the AOR and SSA decreased by 10.57% and 48.76%. The film thickness of modified CDAP particles was more uniform, and the release period was 5.26 times longer compared with the un-modified CDAP with the same coating content. The blending application of CDAP and normal P fertilizer significantly improved the maize grain yield and PUE along with significant increase in enzyme activities. Water-based polishing provided an effective method to improve fertilizer efficiency, and this low-cost technology could be extended to relevant fields.

## Data Availability

Data generated during the current study are available from the corresponding author on reasonable request.
